# Success Factors of Growth-Stage Digital Health Companies: Systematic Literature Review

**DOI:** 10.2196/60473

**Published:** 2024-12-11

**Authors:** Estelle Pfitzer, Laura Bitomsky, Marcia Nißen, Christoph Kausch, Tobias Kowatsch

**Affiliations:** 1 Centre for Digital Health Interventions School of Medicine University of St. Gallen St. Gallen Switzerland; 2 MTIP AG Basel Switzerland; 3 Centre for Digital Health Interventions Institute for Implementation Science in Health Care University of Zurich Zurich Switzerland; 4 Centre for Digital Health Interventions Department of Management, Technology, and Economics ETH Zürich Zurich Switzerland

**Keywords:** digital health, health information technology, success factors, systematic literature review, growth-stage companies, clinical softwares, EHR, electronic health records, health companies, funding, digital therapeutics, substance use disorder, stakeholders

## Abstract

**Background:**

Over the past decade, digital health technologies (DHTs) have grown rapidly, driven by innovations such as electronic health records and accelerated by the COVID-19 pandemic. Increased funding and regulatory support have further pushed the sector’s expansion. Despite early success, many DHT companies struggle to scale, with notable examples like Pear Therapeutics and Proteus Digital Health, which both declared bankruptcy after initial breakthroughs. These cases highlight the challenges of sustaining growth in a highly regulated health care environment. While there is research on success factors across industries, a gap remains in understanding the specific challenges faced by growth-stage DHT companies.

**Objective:**

This study aims to identify and discuss key factors that make growth-stage DHT companies successful. Specifically, we address three questions: (1) What are the success factors of growth-stage digital companies in general and (2) digital health companies in particular? (3) How do these success factors vary across DHTs?

**Methods:**

Following established PRISMA (Preferred Reporting Items for Systematic Reviews and Meta-Analyses) guidelines, a systematic literature review was conducted to answer the questions. A comprehensive literature search was conducted using management and medical literature databases: EBSCO, ProQuest, PubMed, Scopus, and Web of Science. The review spanned scientific articles published from 2000 to 2023, using a rigorous screening process and quality assessment using the Critical Appraisal Skills Programme (CASP) checklist.

**Results:**

From the 2972 studies initially screened, 36 were selected, revealing 52 success factors. We categorized them into internal factor categories (Product and Services, Operations, Business Models, and Team Composition) and external factor categories (Customers, Health Care System, Government and Regulators, Investors and Shareholders, Suppliers and Partners, and Competitors). Of the 52 factors, 19 were specific to DHT companies. The most frequently cited internal success factors included financial viability (n=18) and market demand and relevance of the product and service (n=13). External success factors emphasized the regulatory environment and policy framework (n=15). Key differences were observed between DHTs and broader digital companies in areas such as data security (*P*=.03), system interoperability (*P*=.01), and regulatory alignment (*P*=.02), with DHTs showing a higher frequency of these factors. In addition, success factors varied across different DHT categories. Health System Operational Software companies emphasized affordability and system integration, while Digital Therapeutics prioritized factors related to government regulations and regulatory approval.

**Conclusions:**

Essential characteristics contributing to the success of growth-stage digital health companies have been identified. This work, therefore, fills a knowledge gap and provides relevant stakeholders, including investors and entrepreneurs, with a valuable resource that can support informed decision-making in investment decisions and, in turn, enhance the success of fast-growing digital health companies. In addition, it provides the research community with a direction for future studies, enhancing the understanding, implementation, and growth of DHTs.

**International Registered Report Identifier (IRRID):**

RR2-10.1101/2024.05.06.24306674

## Introduction

Digital health technologies (DHTs), including health system clinical software like electronic health records, have seen remarkable growth in the past decade. Their adoption in US hospitals surged from 7% in 2009 to over 81% by 2019 [1]. The COVID-19 pandemic further accelerated the adoption of DHTs, pushing for considerable innovation and prompting supportive regulatory changes [2]. At the same time, there was a surge in investor interest, with funding for digital health companies increasing more than tenfold [3,4], driving the development of solutions promising to revolutionize modern medicine by making health care more accessible, efficient, and cost-effective [5,6].

Yet, within the dynamic realm of digital health, numerous companies struggle to maintain their initial momentum, failing to progress beyond early-stage adopters despite being initially hailed as industry unicorns. One example includes Pear Therapeutics Inc., a once pioneering firm that gained US Food and Drug Administration (FDA) approval for its standalone digital therapeutics (DTx) solution, reSET, in September 2017. This prescription DTx, which aimed at addressing substance use disorder in tandem with outpatient therapy [7], helped Pear Therapeutics secure substantial funding, pushing its valuation to over US $1.5 billion [8] and earning a position as a leader among DTx companies [9]. Nevertheless, in 2023, Pear Therapeutics Inc declared bankruptcy, providing a cautionary tale for the sector at large. Similarly, Proteus Digital Health Inc.’s bankruptcy in 2020 sent ripples through the industry, particularly after achieving the landmark development of the first FDA-approved “smart pill” that could monitor medication intake and assess its effectiveness. Despite its groundbreaking technology earning a valuation of US $1.5 billion and the proof of concept supported by studies and clinical trials [10], Proteus struggled to scale up and secure a significant market presence. The lesson here is clear: the most revolutionary DHTs cannot significantly impact people if they fail to align with market dynamics and stakeholders’ priorities.

Previous research has examined success factors via literature reviews across a range of industries, including agile transformations [11], product innovation [12,13], and IT companies [14]. However, a notable gap remains concerning growth-stage digital companies, particularly within the digital health sector, with a particular absence of comprehensive literature reviews.

The “fail fast, fail often” philosophy common among technology companies is often impeded by the complex regulatory environment of the health care industry. This cultural clash is further intensified by the deliberate, step-by-step, and lengthy process of health care innovation, which is rooted in the risk-averse clinical principle of “first, not harm” [6,15]. This highlights the need for a study specific to digital health. A thorough analysis of success factors would not only guide investors and entrepreneurs toward informed decision-making in investments and strategy but also enable the digital health research community to develop a theoretical framework of these factors. This unique contribution, in turn, could pave the way for DHTs to have a higher likelihood of success and a broader implementation within the health care system [16,17].

Our study, therefore, seeks to address this gap by examining success factors of growth-stage digital companies in general, with a particular focus on companies offering DHTs. Growth-stage refers to businesses beyond the startup or existence phase and now focusing on expansion and dealing with the complexities of scaling and establishing a competitive market presence [18]. In addition, digital refers to companies that use technologies as their main product or service. Instead of confining ourselves to a single definition of success, we explored a range of interpretations across various studies, highlighting the diversity in understanding success. Building on prior research [14,19-24], we will identify factors that are directly related to companies’ success. To this end, we formulate the following research questions (RQs):

RQ1: What are the general success factors of growth-stage digital companies?

RQ2: What are the specific success factors of growth-stage digital health companies?

RQ3: How do digital health companies’ success factors differ across different DHT categories?

## Methods

### Overview

To answer our RQs, we undertook a systematic literature review. The study was preregistered on the Open Science Framework Registry on August 21, 2023, and the protocol was published on medRxiv [25]. We followed Snyder and Tranfield’s established methodological guidelines [26,27] to create evidence-informed management knowledge, incorporating appropriate elements from the PRISMA (Preferred Reporting Items for Systematic Reviews and Meta-Analyses) statement (Multimedia Appendix 1) [28].

### Eligibility Criteria

Table 1 specifies our inclusion and exclusion criteria, focusing on studies from 2000 to 2023 to capture the significant shifts post-Dot-Com Bubble—a time marking the entry of numerous digital firms into the market [29].

**Table 1:** Inclusion and exclusion criteria for study selection.

Criteria	Inclusion criteria	Exclusion criteria
Research focus	Studies that identify the success factors in digital growth-stage companies	Studies that do not show research methodology, analysis, or discussion. Research on companies without a digital or tech-enabled component.
Publication type	Peer-reviewed academic literature	Gray literature (eg, news articles, company publications, annual reports, studies by nongovernmental organizations, presentations, catalogs)
Year	Only studies from 2000 and onward are considered	Studies published before 2000
Language	Only English studies are considered	Studies published in languages other than English

### Information Sources and Search Strategy

To conduct a thorough search, we used the following databases: (1) EBSCO and ProQuest: renowned and widely used in business research, these databases provide a rich collection of business-related journals, articles, and reports; (2) PubMed: specializing in biomedical and life sciences research, PubMed offers valuable insights at the intersection of health care and business; And (3) Scopus and Web of Science: these multidisciplinary databases cover a broad array of subject areas, ensuring extensive coverage of our topic.

By exploring these databases, we will collect a diverse range of sources covering business-specific research, health care-business intersections, and interdisciplinary perspectives, ensuring a comprehensive search for our study.

To identify studies that focused on success factors in growth-stage digital companies, we used specific search terms centered around the keywords “success factor” and its synonyms indicating high performance, “growth-stage” to ensure we targeted studies on companies with an established product or service, “companies,” and “digital.” For RQ2 and RQ3, we added “digital health” to uncover success factors specific to the DHT sector. We used these search terms to navigate the databases mentioned above, focusing on titles, abstracts, and keywords. By including synonyms, abbreviations, alternative spellings, and related topics, we ensured a thorough search (Multimedia Appendix 2). This targeted approach helped us to identify relevant literature [27], ensuring a comprehensive and systematic collection of studies for our review.

### Study Records

#### Data Management

Following comprehensive database searches, we imported all citations into Mendeley (version 2.91.0) for efficient management. Mendeley served as our secure repository for all data and documents, preserving data integrity and supporting collaborative efforts.

#### Selection Process

Co-authors EP and LB independently reviewed titles and abstracts using Rayyan to assess eligibility, with EP retrieving full texts for closer evaluation. To mitigate selection bias, paired authors examined these texts, resolving any differences through discussion or consultation with the co-author TK. The selection was meticulously recorded, including a PRISMA flow diagram to detail exclusions. Publications selected for RQ1, which pertain to digital health, informed RQ2 and RQ3. As described above, this secondary selection process was conducted systematically and involved 2 independent reviewers.

#### Data Items and Synthesis

To ensure uniform and precise data extraction, we designed a detailed template to record key characteristics of each study, such as the first author, publication year, methodological details, and definitions of success.

For data analysis, the studies were saved into PDFs to facilitate line-by-line coding. This process enabled us to identify and systematically categorize key data points and recurring themes. Following parts of the active categorization framework of Grodal et al [30], we refined our initially identified categories by dropping, merging, and splitting categories. This iterative process led to the creation of a dynamic list of success factors, which was continuously updated and refined through repeated coding processes. These refined categories were then synthesized into higher-order constructs, forming second-order categories.

To enhance our analysis of success factors, we segmented them into internal and external factors, also referred to as third-order categories, thereby differentiating between elements within a company’s control and those outside its direct sphere of influence but are nonetheless essential for strategic planning. Our rationale for distinguishing between internal and external factors comes from foundational management theory [31,32], which suggests that an organization’s success and sustained competitive advantage are influenced by both its internal capabilities and its environment (ie, external factors).

For RQ3, which aims to identify DHT category–specific success factors, we used the DTx Alliance DHT ecosystem. This ecosystem contains the non–patient-facing Health System Operational Software—the software supporting nonclinical systems like operations and finance—and the Health System Clinical Software, which provides clinical support for managing patient care. In addition, patient-facing solutions include Health and Wellness, Patient Monitoring, Care Support, Digital Diagnostics, and Digital Therapeutics. The categorization of DHT-related papers into these specific categories was carried out by two independent authors who resolved any discrepancies through discussion.

### Risk of Bias and Quality Assessment

The Critical Appraisal Skills Programme (CASP) checklist for qualitative research was used to assess the quality of our selected qualitative studies, aiming to discern and evaluate any methodological limitations impacting the research outcomes [33]. Endorsed by reputable organizations like Cochrane and the World Health Organization, CASP’s effectiveness in qualitative evidence synthesis is well-recognized [33-35]. This tool methodically probes a study’s strengths and weaknesses across 10 specific questions, addressing everything from the clarity of objectives to the study’s overall impact and contribution. Using the CASP framework, responses were categorized as “Yes,” “Can’t tell,” or “No,” with studies requiring at least a 70% “Yes” rate to be included in our analysis.

## Results

### Study Selection

**Figure 1:**
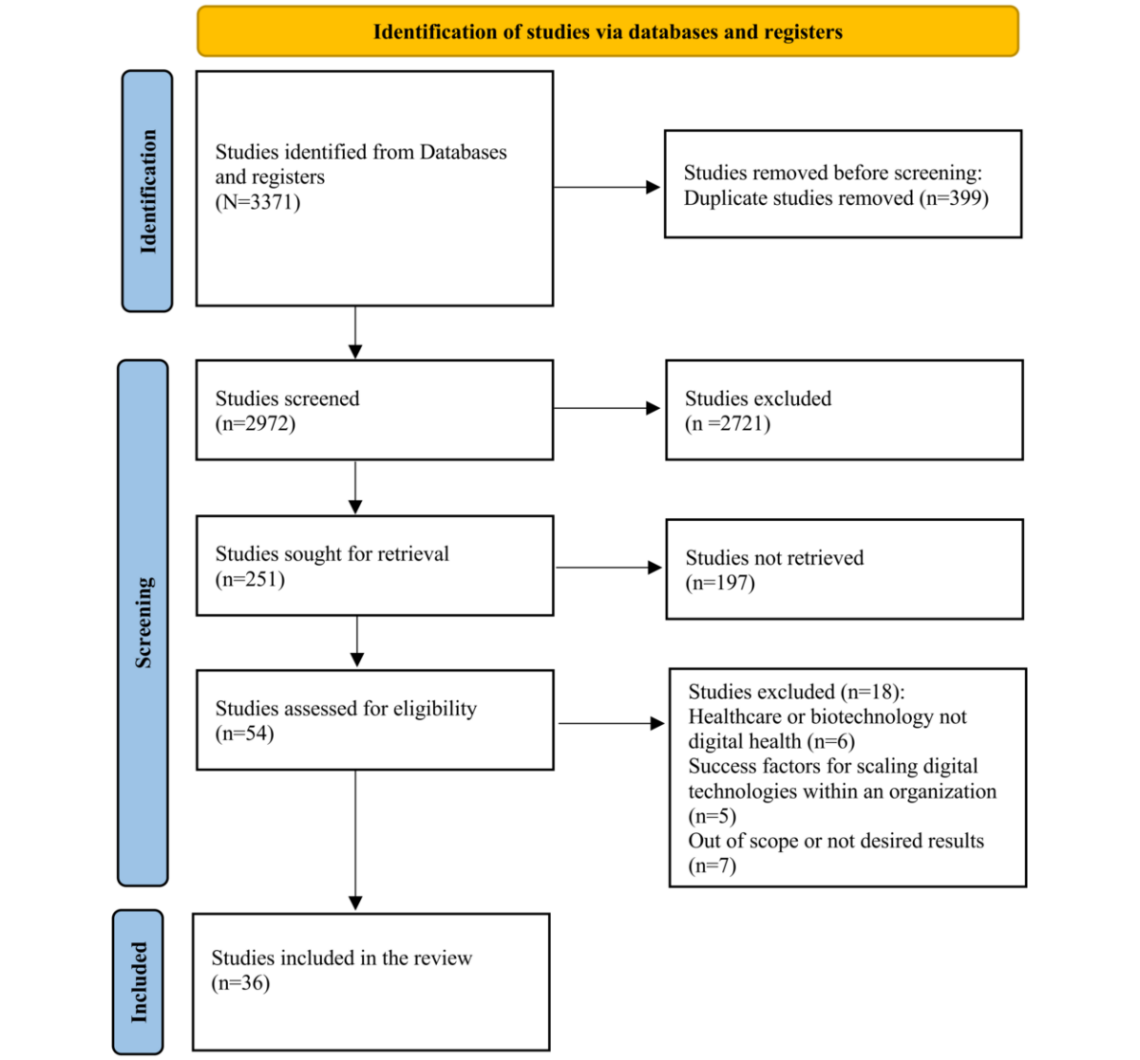
PRISMA (Preferred Reporting Items for Systematic Reviews and Meta-Analyses) flow diagram of the review search, selection, and inclusion process.

Our comprehensive review identified 36 relevant studies from an initial pool of 2972 unduplicated studies. During the initial screening phase, 2721 articles were excluded based on our predefined inclusion and exclusion criteria. We then conducted a detailed screening of the abstracts for the remaining 251 publications, resulting in the further exclusion of 197 articles that did not meet our inclusion and exclusion criteria (Figure 1). This selection followed the assessment of 54 full-text papers for their eligibility, out of which 15 were excluded due to misaligned focus: 6 studies examined non–digital-related companies, while five concentrated on the internal scaling of software projects rather than the broader scope of growing and operating digital enterprises. Predominantly, the studies used qualitative research methods (25/36, 69%), including interviews (8/36, 22.2%) and case studies (10/36, 27.7%). Among the quantitative studies included (11/36, 30.6%), surveys were the primary method (8/36, 22.2%) used to investigate the success factors of growth-stage digital companies.

Of the 36 studies included, 15 are related to DHT (15/36, 41.7%), while the remaining studies describe success factors of companies in other digital sectors such as e-commerce [36-38], e-media [39], and digital companies without a specific sector focus [40-44]. We extracted between 2 and 20 success factors per paper, with a median count of 7 (IQR 5-9.25). Table 2 presents a summary of selected studies, including the publication year, title, research sector, methods used to identify success factors, and how success is defined in each study.

**Table 2:** Summary of selected studies on success factors of digital companies, including the first author, year of publication, title, sector, methods used, and definition of success

Study	Year of publication	Title	Sector	Method characteristics	Success definition
Abel et al [45]	2008	From technology imitation to market dominance: the case of iPod	Other digital	Single case study	Establish industry leadership
Azevedo et al [46]	2022	Scaling-up digital follow-up care services: collaborative development and implementation of remote patient monitoring pilot initiatives to increase access to follow-up care	DHT^a^	Multiple case studies	The scalability and implementation of remote patient monitoring services
Chakrabarti et al [47]	2022	Scaling an internet of things start-up: can alliance strategy help?	Other digital	Single case study	Reach US $10 million revenue in the next 4 years
Chakraborty et al [48]	2023	Critical success factors of startups in the e-health domain	DHT	Interviews	Reach the under-served market by reducing health care costs and increasing service delivery speed
Chanakira [49]	2009	Determining critical success factors of international expansion in African telephony industry	Other digital	Surveys	International expansion in the mobile telephony industry
Cresswell et al [50]	2013	Ten key considerations for the successful implementation and adoption of large-scale health information technology	DHT	Qualitative analysis and literature review	Effective integration and adoption of health IT in large-scale settings
Dong et al [51]	2022	Performance prediction of listed companies in smart health care industry: based on machine learning algorithms	DHT	Machine learning algorithms	Business performance of listed smart health care companies
Feindt et al [36]	2002	Identifying success factors for rapid growth in SME^b^ e-commerce	Other digital	Multiple case studies	Successful competitive performance for the organization
Gengatharen and Standing [37]	2005	A framework to assess the factors affecting success or failure of the implementation of government-supported regional e-marketplaces for SMEs	Other digital	Literature review and multiple case studies	Best practices, theoretical frameworks, and models in the area of e-marketplaces
Greenhalgh et al [52]	2017	Beyond adoption: a new framework for theorizing and evaluating nonadoption, abandonment, and challenges to the scale-up, spread, and sustainability of health and care technologies	DHT	Hermeneutic systematic review and empirical case studies	Achieving large-scale, sustained adoption
Halminen et al [53]	2021	Demonstrating the value of digital health: guidance on contextual evidence gathering for companies in different stages of maturity	DHT	Implementation of WHO^c^’s digital health evaluation guide and CIMO^d^ framework	The appropriate level of evidence required to assess what digital health intervention companies develop
Hughes et al [54]	2021	Scaling digital health innovation: developing a new “service readiness level” framework of evidence	DHT	Interviews	Scaling up DHIs^e^, expanding pilot projects for broader implementation and scalability
Kassner et al [55]	2023	The PropTech investors’ dilemma – what are the key success factors that secure survival?	Other digital	Cox proportional hazards model with nonlinear splines	Survival over a certain period of time
König et al [40]	2023	Evaluation of tech ventures’ evolving business models: rules for performance-related classification	Other digital	Principal component analysis and rough set theory mixed method	Turnover > €300,000 (US $316,000) and tripled the turnover when initially between €100,000 (US $106,000) and €500,00 (US $527,000) and doubled when initially above €500,000 (US $527,000)
Lennon et al [56]	2017	Readiness for delivering digital health at scale: lessons from a longitudinal qualitative evaluation of a National Digital Health Innovation Program in the United Kingdom	DHT	Longitudinal qualitative evaluation using interviews, focus groups, and surveys	Actions required in order to prepare the market and accelerate uptake
Li et al [41]	2022	Founders’ creativity, business model innovation, and growth	Other digital	Surveys and empirical analysis	New venture growth and sustainable competitive advantage
Liu et al [57]	2021	The critical factors affecting the deployment and scaling of healthcare AI: viewpoint from an experienced medical center	DHT	Literature review and case observation	Enhancing medical centers income, reducing costs, and improving care quality
Mishra and Sharma [58]	2022	Digital transformation evaluation of telehealth using convergence, maturity, and adoption	DHT	Multiple case studies	Analyzing telehealth providers, identifying areas for improvement, and contributing to theory development
Ng et al [42]	2017	Exploring performance drivers for technology-based ventures from early stage to expansion: perspectives of venture capitalists	Other digital	Survey and interviews	Technology-based ventures’ development and growth
Nirjar [59]	2008	Innovations and evolution of software SMEs: exploring the trajectories for sustainable growth	Other digital	Survey and model development	The sustained performance of entrepreneurial enterprises [...] in the software sector
Prodan et al [60]	2022	Success factors for scaling up the adoption of digital therapeutics towards the realization of P5 medicine	DHT	Literature review, interviews, and workshop	Scale-up of P5 medicine (personalized, participatory, predictve, precision medicine)
Raravi et al [61]	2013	Critical success factors: service industries	Other digital	Surveys, statistical analysis with IBM SPSS Statistics 19	Successful competitive performance for the individual, department, or organization
Richter and Harst [62]	2021	Tackling the scaling-up problem of digital health applications	DHT	Editorial	Strategies for successfully and sustainably implementing digital health solutions while considering barriers
Rohn et al [63]	2021	Digital platform-based business models–an exploration of critical success factors	Other digital	Semistructured interviews and qualitative content analysis	The configurations under which these business models (digital platform–based business models) are successful and ought to outperform traditional business models
Santisteban and Mauricio [14]	2017	Systematic literature review of critical success factors of IT startups	Other digital	Systematic literature review	The growth of the company and the number of jobs generated
Schlieter et al [17]	2022	Scale-up of digital innovations in healthcare: expert commentary on enablers and barriers	DHT	Workshop	Sustainable digital health diffusion
Sista and De [64]	2021	Scaling up smart city logistics projects: the case of the smooth project	Other digital	Qualitative case study, semistructured interviews, and thematic analysis	The ability of a system to improve its scale by aiming to meet the increasing volume demand
Smagalla [65]	2014	The truth about software startups	Other digital	Analysis of financial results and interviews	Rule of 126: US $100 million in revenue and 20% earnings before interest and taxes margins within 6 years of formation
Srinivasan and Venkatraman [66]	2018	Entrepreneurship in digital platforms: a network-centric view	Other digital	Network-centric theory	Positioning products and services within dynamic digital networks
Sweeney et al [67]	2023	Scaling AI-based industry 4.0 projects in the medical device industry: An exploratory analysis	DHT	Literature review and survey	Sustainable and scalable AI^f^-based industry 4.0 projects in the medical device manufacturing industries
TenBrink et al [43]	2017	Turnaround success in high technology growth stage firms	Other digital	Application of life cycle theory to technology-based firms	Actions and firm circumstances that are associated with successful growth stage turnarounds
Tseng et al [68]	2018	Catalyzing healthcare transformation with digital health: Performance indicators and lessons learned from a Digital Health Innovation Group	DHT	Case study	The “quadruple aim” of enhanced patient experience, reduced cost, improved population, health and improved clinician efficiency
Tsironis et al [69]	2017	e-Business critical success factors: toward the development of an integrated success model	Other digital	Quantitative survey, factor analysis, and structural equation modeling	The successful implementation of e-Business
Tsourvakas and Riskos [39]	2018	Emergent success factors for entrepreneurial e-media companies	Other digital	Case study	Unique characteristics of successful e-media business models
Yang et al [38]	2016	Scale, congestion, efficiency, and effectiveness in e-commerce firms	Other digital	Data envelopment analysis	Operational efficiency and economic effectiveness in e-commerce firms, with considerations for scale, technology, and resource allocation
Yin et al [44]	2019	The success factors of Korean global start-ups in the digital sectors through internationalization	Other digital	Single case study	Scale-up their businesses and gain success through global strategy

^a^DHT: digital health technologies.

^b^SME: small and medium scale enterprises.

^c^WHO: World Health Organization.

^d^CIMO: Context, Interventions Mechanisms, Outcomes Framework.

^e^DHI: digital health intervention.

^f^AI: artificial intelligence.

### Success Definition and Success Metrics

In investigating definitions for success within the digital company landscape, our review revealed various approaches. These are categorized into 5 principal themes: industry position and market dominance [36,37,45,63]; financial performance, operational capabilities, and scalability [17,46,53,54,58,60,64]; market expansion and internationalization [44,49]; technological adoption and innovation [50,51,56,57,62,66,68]; and health impact and service delivery [38,39,41,42,48,52,59,61,67,69,70]. These themes represent a comprehensive view of success, from securing market leadership and achieving financial goals to executing scalable operations, broadening market presence, advancing technological innovation, and effecting social change. This variety in success narratives highlighted multiple interpretations of success across different studies and illustrated the importance of assigning relevant and context-specific success factors to different industry sectors.

### RQ1: General Success Factors of Growth-Stage Digital Companies

In addressing RQ1, we identified 52 distinct success factors across 36 papers. These factors are listed in Table 3. An extended version, with quote examples that illustrate the categories, can be found in Multimedia Appendix 3. All factors contribute to the growth and sustainability of digital companies. We divided the success factors into two main categories: “internal,” those within the company’s control that can be optimized, and “external,” those outside the company’s control but still crucial for strategic planning.

**Table 3:** Breakdown of 52 success factors for growth-stage digital companies, categorized by Internal (Product and Services, Business Models, Operations, and Team Composition) and External (Customers, Healthcare System, Government and Regulators, Investors and Shareholders, Suppliers and Partners, and Competitors) factors

Third-order, second-order, and first-order categories (in order)				Rate		Occurrence (references)
**I. Internal**						
	**Product and Services**					
		Market demand and relevance of the product or service	13		[17,37,40,47,50,52,54,55,57,58,62,63,68]	
		User-centered design	10		[42,46,48,50,53,54,58,60,62,63]	
		Product or service innovation	10		[14,36,39,41-43,45,57,59,67]	
		Quality, performance, and brand trust	9		[39,48,54,56,58,59,63,67,68]	
		Data security, intellectual property protection, and ethical considerations	8		[17,46,48,54,56,60,64,67]	
		Convenience and standardization	7		[17,36,37,44,57,58,67]	
		Training and support programs	7		[37,48,50,54,56,61,67]	
		Price sensitivity	4		[36,48,61,64]	
		Number of patents	2		[40,59]	
	**Operations**					
		Integration or technology change management	11		[17,36-38,42,45,50,52,59,67,69]	
		Optimized internal processes	10		[36,37,41,42,45,47,65,67-69]	
		Performance monitoring	9		[42,47,50,53,58,65,67-69]	
		Interoperability	6		[17,50,54,56,60,62]	
		Modern infrastructure	6		[42,48,50,56,64,67]	
		Risk management	5		[14,47,49,53,64]	
		Sales and marketing effectiveness	3		[36,37,65]	
		Workforce management	2		[17,47]	
	**Business models**					
		Financially viable	18		[14,17,37,38,47-49,51-54,57,59,60,63-65,67]	
		Adaptability and flexibility to market changes and disruptions	11		[17,38,41-44,46,48,52,58,61]	
		Use of existing and emerging technologies	9		[17,42,44,47,51,55,58,59,61]	
		Value proposition and differentiation in the market	5		[17,41,47,48,52]	
		Business model consideration at an early stage	5		[17,38,50,59,64]	
		Competitive awareness and strategic positioning	4		[45,48,66,67]	
	**Team composition**					
		Leadership experience and qualities of team members	11		[14,17,37,41-43,48,50,54,63,69]	
		Diversity of skills and expertise within the team	8		[17,38,42,46,48,57,59,68]	
		Innovativeness and technical competence	5		[14,17,42,52,59]	
		Organizational size	4		[40,43,55,70]	
		Experience in the sector	3		[14,41,42]	
		High motivation and focus	3		[14,37,44]	
		Training and development	2		[42,70]	
		Stock options	1		[42]	
**E. External**						
	**Customers**					
		Customer feedback and satisfaction with the product or service	12		[36,39,41,42,47,48,54,61,62,65,67,69]	
		Customers awareness raising	6		[17,37,56,57,60,63]	
		Regional market size, consumer needs, and behavior	5		[14,37,42,49,58]	
		Brand image and community building	2		[36,39]	
	**Health care system**					
		Long-term integration with existing systems	7		[17,46,53,54,56,57,62]	
		Increase in affordability	5		[50,56,60,67,68]	
		Improved diagnosis or care	4		[54,58,60,68]	
		Enhanced coordination	4		[57,62,67,68]	
		Resource allocation and utilization optimization	3		[46,54,60]	
		Enhanced patient experience	1		[68]	
	**Government and regulators**					
		Regulatory environment and policy framework	15		[14,17,37,48,52-54,56-58,60,62,64,67,68]	
		Government endorsement and direct support	3		[14,60,64]	
	**Investors and shareholders**					
		Synergy and alignment of shareholders’ goals and objectives	4		[37,64,66,69]	
		Investor backing and fit	4		[14,40,55,66]	
		Access to resources, networks, and expertise through partnerships	4		[43-45,66]	
	**Suppliers and partners**					
		Alliance strategy in place	7		[14,17,47,49,54,64,66]	
		Collaboration with larger organizations	4		[36,45,66,68]	
		End-to-end value chain involvement	2		[17,46]	
	**Competitors**					
		Market entry timing and organizational maturity	3		[14,45,55]	
		Intensity of competitors in the market	1		[14]	
		Collaborative opportunities with competitors for mutual growth	1		[14]	

### Internal Factors

Internal factors play an important role in shaping the operational and strategic framework of companies. In our comprehensive analysis, we identified 31 internal success factors, which are organized into 4 distinct second-order categories.

Factors within the I.1. Product and Services category emerged as the most prevalent, with a total of 70 occurrences (22.7% of all occurrences) in the reviewed literature. The leading factor in this category, I.1.a. Market Demand and Relevance of the product or service, was identified 13 times (13/70, 18.6%), underscoring the critical need to align products with consumer preferences and market trends. I.1.b. User-Centered Design and I.1.c. Product or Service Innovation were each highlighted 10 times (10/70, 14.3%), emphasizing the importance of prioritizing customer experience in product or service design and of continuously innovating such offerings. Other significant factors identified were I.1.d. Quality and Performance and Brand Trust (n=9, 2.9%), I.1.e. Data Security and Intellectual Property Protection (8/70, 2.6%), and I.1.f. Convenience and Standardization of Offerings (n=7, 2.3%). Together, these highlight the central role of quality, security, and customer-centered design as key drivers of a company’s performance.

The I.2. Business Models category was highlighted 52 times (16.9% of all occurrences), with I.2.a. Financial Viability (18/52, 34.6%) as the most mentioned success factor. This category emphasizes the significance of the I.2.c. Use of Existing and Emerging Technologies (9/52, 17.3%) and a I.2.d. Strong Value Proposition and Differentiation in the Market (5/52, 9.6%), associated with I.2.f. Competitive Awareness (4/52). In addition, I.2.e. Early Business Model Planning (5/52, 9.6%) emerged as another important management practice vital for securing a firm’s long-term viability.

I.3. Operations-related success factors drew 52 statements (16.9% of all occurrences), emphasizing the importance of*_._* seamless I.3.a. Technology Integration and Change Management (11/52, 21.2%) and I.3.b. Optimized Internal Processes (10/52, 19.2%) in fostering operational efficiency. I.3.c. Performance Monitoring (9/52, 17.3%) and I.3.d. Interoperability (6/52, 11.5%) highlighted important management practices, as well as again a key technology requirement. I.3.f. Risk Management (5/52, 9.6%) and I.3.g. Effective Sales and Marketing (n=3/52, 5.8%) also help support the success of digital companies.

With 37 (12.0% of all occurrences) mentions, the I.4. Composition and Dynamics of the Team emerged as another key domain impacting a company’s success trajectory. I.4.a. Leadership Qualities (11/37, 29.7%) and I.4.b. Diversity of Skills Within the Team (8/37, 21.6%) rank high in enabling the success of (digital) enterprises. In addition, I.4.d. Organizational Size (4/37, 10.8%), I.4.e. Sector-Specific Experience (3/37, 8.1%), and I.4.f. High Motivation Levels (3/37, 8.1%) pointed to important internal people-related factors to companies’ success.

### External Factors

The external factors emphasize the interactions of companies with various stakeholders. Overall, we distinguished 21 factors within 6 second-order categories. The most frequently mentioned external category concerns the engagement with E.1. Customers (n=25, 8.1% of all occurrences). E.1.a. Customer Feedback and Satisfaction (12/25, 48.0%) emerged as critical for refining product or service offerings and ensuring alignment with market needs. E.1.c. Understanding Regional Consumer Behavior (5/25, 20%) and E.1.d. Efforts in Brand and Community Building (2/25, 8%) are essential for cultivating customer loyalty and expanding market presence.

Impact on the E.2. Healthcare System was also highlighted as a crucial success factor in DHT-related papers, receiving 24 mentions (7.8% of all occurrences). This category highlights the importance of E.2.a. Long-Term Integration with Existing Systems (7/24, 29.2%), E.2.a. Affordability (5/24, 20.8%), and E.2.c. Improved Care Outcomes (4/24, 16.7%) for health care–related digital solutions.

Alignment with E.3. Government and Regulators is crucial, as raised 18 times (5.8% of all occurrences) in the literature review. The E.3.a. Regulatory Environment and Policy Framework (15/18, 83.3%) plays a fundamental role in shaping the market landscape for digital companies. E.3.b. Government Endorsement and Direct Support (3/18, 16.7%) are essential for navigating key legal and financial hurdles.

The alignment and interaction with other stakeholders, including investors, suppliers, and competitors, were mentioned less frequently. However, E.5.a. Alliance Strategy in Place (7/13, 53.8%) and E.5.b. Collaboration with Larger Organizations (4/13, 30.8%) within E.5. Suppliers and Partners (n=13, 4.3% of all occurrences) surfaced as essential to strengthening the value chain and enhancing product or service offerings, for example, through robust partnerships.

### RQ2: Specific Success Factors of Growth-Stage Digital Health Companies

**Figure 2:**
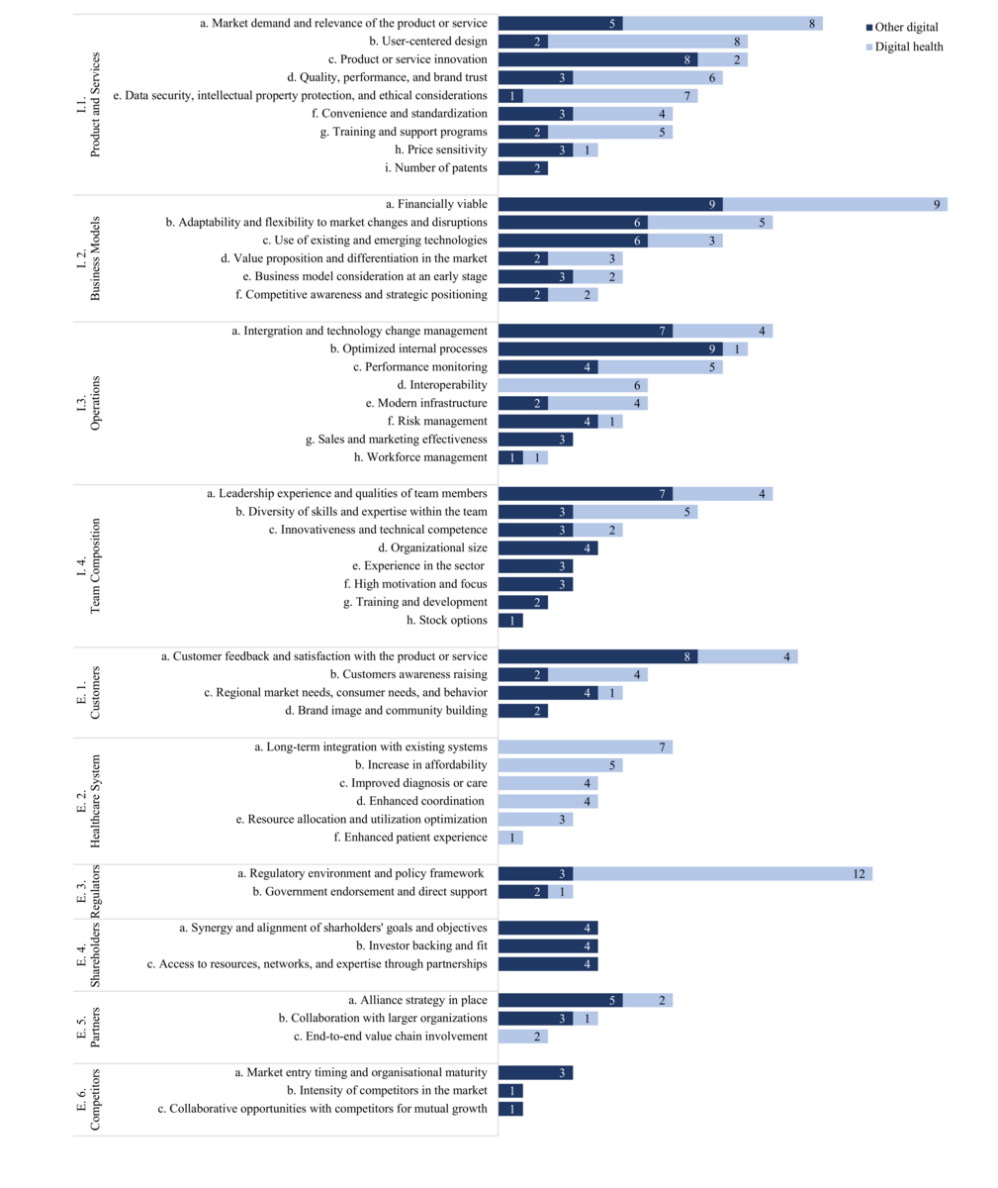
Comparative analysis of success factor occurrences in growth-stage digital health companies versus other digital sectors, categorized by Internal (I) and External (E) factors. Light blue represents success factors in Digital Health companies, while dark blue represents success factors in broader digital industries. The numbers indicate the frequency of occurrences within the studies included in our literature review.

Our analysis examined success factors pertinent to the digital health sector relative to those in broader digital industries (Figure 2). Within the first category I.1. Product and Services, I.1.e. Data Security, Intellectual Property Protection, and Ethical Considerations (n_OtherDigital_=1, n_DigitalHealth_=7; *P*_Fisher exact test_=.03) occurred with more than twice the frequency in digital health literature than in combined mentions within other digital industries underscoring the sector’s unique priorities and challenges associated with handling patient data in the Digital Health sector. In reviewing the I.2. Business Models category, our analysis revealed that the representation of most factors was comparable between health care–focused companies and those in broader digital sectors. Fisher exact test showed no significant differences, with all factors yielding *P*>.05. This suggests similar strategic approaches across these industry segments. The I.3. Operations analysis showed I.3.d. Interoperability considerations (n_OtherDigital_=0, n_DigitalHealth_=6; *P*_Fisher exact test_=.01) as having greater significance to digital health companies. This element is crucial for the seamless functioning within and between established health care infrastructures. In addition, within I.4. Team Composition, the I.4.b. Diversity of Skills and Expertise within the team was notably more prevalent among DHT companies, indicating the necessity for a wide range of competencies to address the multifaceted nature of health care and health technology challenges, though the difference did not reach statistical significance (n_OtherDigital_=3, n_DigitalHealth_=5; *P*_Fisher exact test_=.49).

Regarding the influence of external stakeholders on digital companies, beyond just health care stakeholders, which appear only in DHT-related papers, E.3. Government Bodies and Regulatory Agencies are especially pertinent to digital health companies. All factors within this category show a higher representation in the health care domain, with the E.3.a. Regulatory Environment and Policy Framework as the most frequently mentioned (n_OtherDigital_=3 vs n_DigitalHealth_=12; *P*_Fisher exact test_=.02).

### RQ3: Success Factors Differences Across the DTx Alliance Digital Health Technology Categories

**Figure 3:**
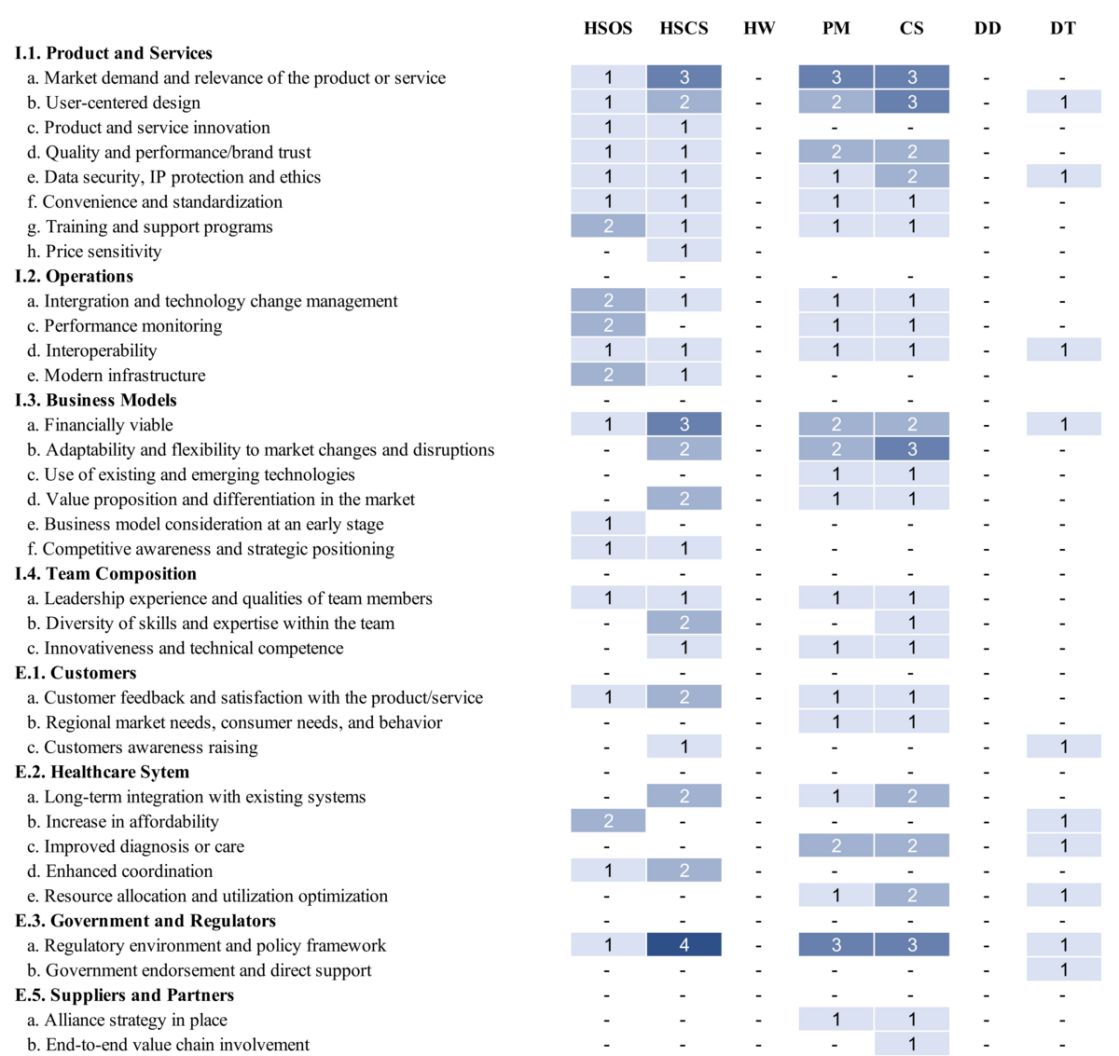
Distribution of success factors across DTx Alliance categories. A “-” symbol indicates that the success factor is not referenced. The shade of blue represents the frequency of occurrence, with the lightest shade signifying one occurrence and the darkest shade indicating 4 occurrences. The categories are as follows: Health System Operational Software, Health System Clinical Software, Health and Wellness, Patient Monitoring, Care Support, Digital Diagnosis, and Digital Therapeutics.

We categorized the included papers focusing on digital health companies (15/36, 41.7%) based on the DHT categories defined by the DTx Alliance [71]: Health System Operational Software (2/14, 14.3%) [50,67], Health System Clinical Software (3/14, 21.4%) [48,57,62], Care Support (3/14, 14.3%) [52,54,58], Patient Monitoring (4/14, 28.6%) [46,52,54,58], and Digital Therapeutics (1/14, 7.1%) [60]. Not all papers were classifiable within these subcategories, as 5 focused on the broader scope of digital health without fitting into distinct segments [17,51,53,56,68]. None of the papers reviewed fell into the categories of Health and Wellness or Digital Diagnostics (Figure 3).

By focusing on factors more commonly mentioned within each category relative to the other 6, we observe that in Health System Operational Software, I.1.g. Training and Support Programs for Products or Services is prominent. In addition, I.2.a. Interoperability and Technology Change Management and I.2.e. Modern Infrastructure were found more often (2/5, 40%) in this category, highlighting their role in maintaining the integrity of operational software in health care systems. Moreover, E.2.b. Increase in Affordability of Health Care (2/3, 66.7%) positively impacts the success of Health System Operational Software.

In the category of Clinical Software, the emphasis on navigating the E.3.a. Regulatory Environment of the Location (4/12, 33.3%) emphasizes the critical nature of regulatory compliance. It is vital for such software not only to serve as a functional tool but to deliver tangible value, underscored by mentions of being I.3.a Financially Viable (3/9, 33.3%) and offering an attractive I.3.d. Value Proposition and Differentiation in the market (2/4, 50%). Effective Health System Clinical Software also depends on E.2.d. Enhanced Workforce Coordination (2/3, 66.6%) within health care environments and a team’s I.4.b. Diversity of Skills and Expertise (2/3, 66.6%), whereby both clinical knowledge and software development proficiency are needed.

For Care Support and Patient Monitoring solutions to be broadly embraced, key aspects like the service’s I.1.d. Quality and Performance and Brand trust (2/6, 33.3%) and E.2.a. Improved Diagnosis or Care (2/5, 40.0%) must be prioritized. Addressing the hesitation to switch from traditional to Telehealth and other innovative technologies involves not only focusing on these critical factors but also integrating I.1.b. User-Centered Design. In addition, the adoption of Digital Care Support by health care providers necessitates that these technologies significantly improve E.2.e. Resource Allocation and Utilization Optimization (2/4, 50%) and be I.3.b. Flexibility to Market Changes and Disruptions (3/7, 42.9%) thereby enhancing the efficacy of the health care system. Only one paper fell into the DTx category, so our insights are limited. However, all the factors related to Government and Regulation were mentioned, underscoring their significance in DTx adoption.

## Discussion

### Theoretical Contribution

Our initial results indicate that, despite numerous advancements in DHT, the body of research specifically focused on the success factors of growth-stage digital health companies remains surprisingly limited. From a pool of 2972 studies, only 36 were relevant, with just 15 targeting DHTs directly. This scarcity of focused research suggests that one contributing factor could be a misalignment between current research priorities and the critical needs of the evolving digital health sector.

Internal success factors for digital companies, including those in the DHT sector, have been identified more frequently than external factors, which may be attributed to the limited control companies have over external influences. Among the internal factors, financial viability emerges as particularly critical. Indeed, the future of these companies is more closely tied to the demand for and proper payment for their products or services than to their contributions to improved health outcomes and more efficient health care provision. While extensive research focuses on the health impact of DHT, understanding their financial implications and assessing their economic value is equally crucial.

Our findings highlight that in external factors for DHT companies, ongoing support from regulatory bodies and policymakers is particularly essential for the continued success of these companies. Such support should include consistent guidance, assistance, and the development of policy frameworks that promote business growth. We urge stakeholders in DHT to clearly articulate the benefits their solutions offer to the health care system and to actively engage with regulatory authorities. This interaction not only ensures compliance but also encourages regulators to recognize and address any existing regulatory deficiencies within the DHT sector. Germany’s adoption of the Digital Healthcare Act and the Digital Health Application Regulation highlights its forward-thinking approach to enhancing the digital health sector. This legislative framework not only accelerates the integration of digital health solutions, like prescription health apps but also sets a precedent for digital health advancement, fostering innovation and ensuring equitable access to digital health services [72]. For DHT companies, maintaining dialogs with public payers and insurance providers is important to ensure appropriate reimbursement. Moreover, adapting existing laws to accommodate new business models and technologies and efforts to ensure market fairness are vital steps in supporting the digital health ecosystem [54,60,73].

Integrating insights from the literature on success factors in other domains, such as product innovation, our study identifies both commonalities and unique aspects pertinent to DHT companies. Like general product innovation, the success factors we identified for DHT emphasize the importance of market demand, robust internal processes, and early customer involvement [12,74,75]. However, unique challenges, such as stringent regulatory compliance and the need for interoperability with existing health systems, set digital health apart from other sectors. While environmentally sustainable product innovation research underscores the importance of interfunctional collaboration and Research and Development investments [13], DHT also demands a heightened focus on data security and patient privacy. In addition, our findings highlight that adaptability and flexibility to market changes and disruptions are particularly crucial for DHT, especially given the regulatory uncertainty [76].

In examining the critical success factors across the various DTx Alliance DHT categories, as highlighted by RQ3, it becomes clear that the digital health sector is diverse, with the different categories having common as well as distinct success factors. For instance, Care Support solutions underscore the vital role of adaptability in the face of external disruptions, such as those brought about by global health crises like the COVID-19 pandemic [77]. This ability to adapt ensures the continuity of care and showcases the agility of DHTs to pivot in response to evolving health needs. To avoid encountering unforeseen and avoidable obstacles, digital health companies must understand the unique challenges and success factors specific to their DHT categories and the overarching factors driving their business forward.

Furthermore, RQ3 points out a notable research gap in the areas of Health and Wellness digital solutions and Digital Diagnostics. There’s a clear need for more studies on best practices within these fields to provide a fuller picture of the critical focus areas for companies based on their DHT applications. Encouraging further research in these under-explored areas could greatly enrich our understanding of success factors in the evolving landscape of DHT.

Our analysis paints a picture of success in the digital health realm as a complex interplay of technological innovation, user-centric design, regulatory engagement, and systemic flexibility, among others. Given this complexity, stakeholders within the digital health ecosystem must adopt a comprehensive approach when developing and deploying DHTs. This means that technologies should push for innovation and be crafted to align with regulatory standards and operational exigencies, optimize business models, and meet market demands and stakeholders’ expectations. By embracing this holistic strategy, digital health initiatives can ensure disruption of our health care landscape and sustained efficacy and impact in enhancing health care delivery.

### Managerial Implications

The findings of this study hold significant implications for the digital health research community, highlighting the need for a deeper and more thorough exploration of success factors in the digital health sector. It advocates for a collaborative effort to narrow the divide between theoretical research and practical application, encouraging researchers to validate and refine identified success factors and to discover new ones, particularly in underresearched areas such as digital health technologies for Health and Wellness (prevention), DTx or Digital Diagnostics. This effort will not only enrich the theoretical framework guiding the development and assessment of DHTs but also improve practical guidelines for their effective implementation and integration into the healthcare system.

This meticulously curated list of success factors is valuable for entrepreneurs and stakeholders active in the digital health industry. This resource can empower them to prioritize and focus on the most influential factors for success in this dynamic and competitive market. Founders in the digital health sector, particularly those with an established product or service and initial paying customers, often face challenges in scaling significantly after gaining initial traction. This research will be invaluable for them, offering targeted guidelines on emphasizing specific strategies, collaborative efforts, and organizational practices to facilitate their transition. They can go through the list one by one, establishing their current position regarding each factor and self-diagnosing areas where they are lagging and need more attention. For example, a company might notice they lack an alliance strategy and can brainstorm potential larger partners who could benefit from or deploy their product, ultimately boosting their growth. It provides a roadmap for leveraging initial successes into broader, sustainable growth within the digital health industry.

Investors in growth-stage digital health companies will find this research helpful for making informed decisions about which businesses have established the right priorities for scaling to success. It offers detailed insights into identifying companies poised for effective expansion. Furthermore, the research provides structured recommendations on scaling strategies, which are especially valuable for investors who serve on the boards of these companies, guiding them in advising digital health businesses toward sustainable growth.

As insurance companies begin implementing DHTs and offering them to their patients [78], this research provides essential guidance for selecting which DHT companies to collaborate with. Insurance providers can leverage the identified success factors to assess the viability of various digital health solutions. By choosing partners that align with these success factors, insurance companies can ensure they are integrating effective and reliable technologies into their offerings. The insights from this study can help insurance companies develop strategic partnerships and implement DHTs that support their goals of delivering high-quality, cost-effective care [79].

### Limitations and Future Research Recommendations

The main limitation of the current work is that the success of DHT can significantly change depending on the health care environment, the demographics of the patient population, and the specifics of local regulations. Such diversity presents challenges universally applying identified success factors to all situations. We recommend that future research on the success of companies should focus on specific geographic regions to provide more tailored guidance.

Another limitation arises when comparing success across different digital health categories. Categories such as Digital Diagnostics or Health and Wellbeing have not been sufficiently analyzed regarding success factors. This lack of research limits our understanding of success factors in these evolving areas of DHTs. Ideally, we would have extensive research for each DTx Alliance category to better understand the key aspects that contribute to success in each sector. This would allow companies to receive tailored recommendations specific to their DHT category. Therefore, it is essential for researchers specialized in specific DHTs to conduct further studies to identify and elaborate on the critical and category-specific success factors.

Despite thorough attempts to search relevant literature extensively, the selection process, informed by precise inclusion and exclusion criteria, could have led to bias in the outcomes. Our search was limited to peer-reviewed literature to maintain high academic rigor and reliability, which excluded industry reports and gray literature. While we acknowledge that incorporating gray literature could yield additional insights, our aim for this systematic review was to focus on peer-reviewed sources to ensure the highest quality of included papers. We systematically screened papers, diligently identifying and coding quotes that revealed success factors. We categorized these factors based on our best judgment, acknowledging the nuanced interrelations among them. It is important to note that another group might have selected slightly different success factors; however, our use of broader categories ensures a comprehensive overview.

In addition, the rapid evolution of DHT might outstrip existing research, risking the obsolescence of identified success factors as innovations and updates arise. This dynamic could also reveal new factors not identified in this study. Therefore, we suggest future researchers in this field use our comprehensive list as a baseline to identify which success factors have become outdated or have emerged as new.

### Conclusion

This systematic review explored success factors critical for growth-stage DHT companies, highlighting the interplay between internal and external factors such as market demand, regulatory compliance, and financial viability. Significant gaps were identified, notably in the under-represented categories like Digital Diagnostics, pointing to the need for focused research. The review emphasizes the necessity of adaptive strategies and innovation to meet changing market and regulatory demands. This study not only enhances academic understanding but also offers practical insights for stakeholders in the digital health industry, providing recommendations for DHT companies.

## Appendix

Multimedia Appendix 1 PRISMA (Preferred Reporting Items for Systematic Reviews and Meta-Analyses) checklist.

Multimedia Appendix 2 Detailed search terms based on alternative keywords.

Multimedia Appendix 3 Detailed examples for each 1st-order category, illustrating thematic synthesis with quotes from relevant studies.

## Abbreviations

CASP: Critical Appraisal Skills Programme
DHT: digital health technologies
DTx: digital therapeutics
FDA: Food and Drug Administration
PRISMA: Preferred Reporting Items for Systematic Reviews and Meta-Analyses
RQ: research questions

## Data Availability

The data supporting this study’s findings are available from the corresponding author upon request. The research protocol and other relevant materials can be provided.
