# Supplementary material for: Success Factors of Growth-Stage Digital Health Companies: Systematic Literature Review
Source: J Med Internet Res. 2024 Dec 11;26:e60473. doi: 10.2196/60473 (PMC11669886; doi:10.2196/60473)
Supplement: Multimedia Appendix 2 [file jmir_v26i1e60473_app2.docx]

**Multimedia Appendix 1: Search strategy**

Table S1: Search terms based on alternative keywords.

| **NB** | **Keyword category** | **Search terms** |
| --- | --- | --- |
| **I** | Success Factors | Success factors* OR Key success factors* OR Critical success factors* OR Factors contributing to success* OR Determinants of success* OR Drivers of success* OR Elements of success* OR Factors influencing success* OR Achieving success* OR Factors for successful outcomes* OR Achieving desired outcomes* OR Success determinants* OR Successful outcome* OR Predicted outcome* OR Outcomes of success* OR Desired outcome* OR Outcome measurement* OR Outcome evaluation* OR Outcome assessment* OR High performance* OR Performance* OR Success prediction* |
| **II** | Growth-stage | Growth stage* OR Development stage* OR Expansion stage* OR Scale-up stage* OR Scale-up* OR Scaling* OR High-growth stage* OR Rapid growth stage* OR Growth-oriented* OR Growing companies* OR Maturing companies* OR Evolving companies* OR Advancing companies* OR Progressive companies* OR Progressing* |
| **III** | Companies | Companies* OR Firms* OR Organizations* OR Enterprises* OR Ventures* OR Startups* OR Businesses* OR Industry* |
| **IV** | Digital | Digital* OR Digitization* OR Digitalization* OR Digital transformation* OR Digital solutions* OR Digital technologies* OR Digital tools* OR Digital systems* OR Digital processes* OR Digital capabilities* OR Digital strategies* OR Digital adoption* OR Digital implementation* OR Technology-driven* OR Tech-enabled* OR Tech-oriented* OR Tech-driven* OR Technology-focused* OR Software* OR Software solution* OR Automation* OR E-commerce* OR Internet of Things* OR Cloud computing* OR Data analytics* OR Cybersecurity* OR Artificial Intelligence* OR AI* OR Machine Learning* OR ML* OR Mobile apps* OR Web applications* OR User experience* OR Platform* OR Connectivity* OR Digital innovation* OR Digital disruption* |
| **V** | Digital Health | Health* OR Medicine* OR Digital health* OR Health technology* OR Medical technology* OR Health IT* OR Healthcare technology* OR Health informatics* OR Telemedicine* OR Telehealth* OR Health apps* OR Wearables* OR E-health* OR M-health* OR Health innovation* OR Healthtech* OR MedTech* OR Health data* OR Health analytics* OR Health software* OR Health platforms* OR Medical informatics* OR Healthcare informatics* OR Digital medicine* OR Remote patient monitoring* OR Virtual healthcare* OR Health monitoring technology* OR Mobile health application* OR Health data management* OR Wearable health device* OR Digital therapeutic* OR Health information system* OR Healthcare informatic* OR Remote health monitoring* OR Electronic health records* OR Connected health devices* OR Health tech advancements* OR Health technology integration* OR Health technology adoption* OR Health technology assessment* OR Health technology implementation* OR Healthcare software solution* OR Mobile medical application* |
