# Supplementary material for: Success Factors of Growth-Stage Digital Health Companies: Systematic Literature Review
Source: J Med Internet Res. 2024 Dec 11;26:e60473. doi: 10.2196/60473 (PMC11669886; doi:10.2196/60473)
Supplement: Multimedia Appendix 3 [file jmir_v26i1e60473_app3.docx]

**Multimedia Appendix 2: Thematic Synthesis**

Table S2: Success factors of growth-stage digital companies with quotes to showcase thematic synthesis

| **3^rd^ order categories** | **2^nd^ order**  **categories** | **1^st^ order**  **categories** | **Illustrative quote** | |
| --- | --- | --- | --- | --- |
| **I. Internal** | 1. **Product & Services** | 1. Market demand and relevance of the product/service | | *Evidence is needed to answer "does it solve or contribute to our real challenge in the system [...] so people see its value?"* (53) |
|  |  | 1. User-centered design | | *A system should be both fit for organizational purpose and fit for clinical practice. There are countless examples of systems that have been procured but never used* (58) |
|  |  | 1. Product/service innovation | | *Health care system is changing rapidly. […]. We have to improve our system regularly to deal with the current need and future opportunities* (65) |
|  |  | 1. Quality and performance/brand trust | | *In the value delivery dimension, the increase of transaction speed and efficiency through latest digital technologies could be quoted as one of the most crucial factors* (50) |
|  |  | 1. Data security, intellectual property protection and ethical considerations | | *Security risks within the scope of AI project implementation may not be overlooked to ensure high security, data integrity and compliance with the regulatory standards* (69) |
|  |  | 1. Convenience and standardization | | *Since clinical work is already complex on its own, AI products should be easy to install and use* (61) |
|  |  | 1. Training and support programs | | *Training and technical support can be critical if technology is complicated* (41) |
|  |  | 1. Price sensitivity | | *Buy-In & Tariffs were found to be the other most significant contributor of organizational growth among all the critical success factors* (67) |
|  |  | 1. Number of patents | | *Other researchers […] have also considered the number of patents generated by a firm (copyrights in case of software firm) to be the most reliable indicator* (66) |
|  | 1. **Operations** | 1. Integration/technology change management | | *Compatibility with users’ systems promotes participation […] by improving efficiency and lowering costs* (41) |
|  |  | 1. Optimized internal processes | | *Efficiently use the resources and capabilities of the enterprise* (45) |
|  |  | 1. Performance monitoring | | *The first factor, 'Performance Measurement and Improvement,' involves performance analysis in all areas of a company, encouraging improvement and innovation and creating value for the company* (70) |
|  |  | 1. Interoperability | | *Technical and service interoperability needs to be prioritized and, if necessary, incentivized to ensure the scaling up of digital health care across systems and sectors* (60) |
|  |  | 1. Modern infrastructure | | *Developing the right infrastructure is an essential part of planning activity* (58) |
|  |  | 1. Risk management | | *Conducting the relevant studies during the pilot stage is crucial to […] visualize potential risks* (56) |
|  |  | 1. Sales and marketing effectiveness | | *It’s about developing a finely honed, repeatable sales message that will resonate with customers. Only after this has been achieved can a software firm effectively grow both its sales and its sales organization* (74) |
|  |  | 1. Workforce management | | *The challenge was to upskill them (employees) and retain them* (72) |
|  | 1. **Business Models** | 1. Financially viable | | *After analysing the performance of listed companies in the smart healthcare industry, we find that there are also many problems in the development process of the companies, and the financial risks in its development are relatively large* (59) |
|  |  | 1. Adaptability and flexibility to market changes and disruptions | | *The reality of any technology implementation project is that at an empirical level they are inextricably interlinked and dynamically evolving, often against a rapidly shifting policy context* (71) |
|  |  | 1. Use of existing and emerging technologies | | *The factor of convergence offers a direction for fast growth, by connecting to the existing platform or recombining existing resources for creating a new product* (48) |
|  |  | 1. Value proposition and differentiation in the market | | *Providing added value (the degree to which the digital innovation can address the needs or ease the pains of users)* (19) |
|  |  | 1. Business model consideration at an early stage | | *Therefore, the definition of a clear vision is necessary to test that the designed model practically works, and that the underlying system is in place* (56) |
|  |  | 1. Competitive awareness and strategic positioning | | *Entrepreneurs in digital platforms increase their chance of scale-up success by reducing the overlap of their modules with those of competitors in their module networks* (63) |
|  | 1. **Team Composition** | 1. Leadership experience and qualities of team members | | *Having the right people for crucial roles is critical as research suggests that 5% of the positions drive 95% of the transformational value* (69) |
|  |  | 1. Diversity of skills and expertise within the team | | *The interdisciplinary co-creation is an enabler for scaling up digital solutions* (51) |
|  |  | 1. Innovativeness and technical competence | | *Technological and managerial skills, aptitudes and knowledge required to gain competitive advantage* (68) |
|  |  | 1. Organizational size | | *It is considered that the bigger the size of the entrepreneurial team, the greater the talent* (68) |
|  |  | 1. Experience in the sector | | *Founders and senior management’s experience in industry* (46) |
|  |  | 1. High motivation/focus | | *Motive affects market mechanism and services offered, benefits and success and the time taken to achieve it* (41) |
|  |  | 1. Training and development | | *Each of the other indicators in Human Capital obtained a higher average score in the expansion stage […], especially for continuing training and development and multidisciplinary teams* (46) |
|  |  | 1. Stock options | | *The use of stock options to improve the alignment of interests* (46) |
| **E. External** | 1. **Customers** | 1. Customer feedback and satisfaction with the product/service | | *Interviews revealed that successful companies often obtained customer feedback early in beta testing and only invested in features that customers needed* (74) |
|  |  | 1. Customers awareness raising | | *There is a need to invest in further awareness raising, upskilling of consumers* (60) |
|  |  | 1. Regional market size, consumer needs and behaviour | | *The most important market issue is market size in terms of population of the destination country* (57) |
|  |  | 1. Brand image and community building | | *The ability to build up a brand […] using online and offline brand-building techniques* (40) |
|  | 1. **Healthcare System** | 1. Long term integration with existing systems | | *Successful implementation of digital health application into existing systems (meso level) is an outcome which is not sufficiently considered* (62) |
|  |  | 1. Increase in affordability | | *All explored countries and regions have additional requirements related to […] health economic implications* (55) |
|  |  | 1. Improved diagnosis or care | | *Fundamental pieces of evidence on clinical efficacy and patient safety were key* (53) |
|  |  | 1. Enhanced coordination | | *Health systems are seeking to leverage innovative DH technologies to promote […] improved clinician efficiency* (64) |
|  |  | 1. Resource allocation and utilization optimization | | *The healthcare system should provide necessary information, especially on costs to the DTx provider for the benefit of both parties* (55) |
|  |  | 1. Enhanced patient experience | | *Health systems are seeking to leverage innovative DH technologies to promote the 'quadruple aim' of enhanced patient experience* (64) |
|  | 1. **Government & Regulators** | 1. Regulatory environment and policy framework | | *NHS England created specific programs to enable rapid uptake of digital innovations* (55) |
|  |  | 1. Government endorsement and direct support | | *Government support and incentives - Tax breaks for participation/e-business training and consultation could positively affect uptake* (41) |
|  | 1. **Investors & Shareholders** | 1. Synergy and alignment of sharholders' goals and objectives | | *Vision needs to be accepted by various stakeholders* (56) |
|  |  | 1. Investor backing and fit | | *The authors find that in addition to the size, financing condition plays the most critical role […], including the number of financing rounds and maximum number of investors over lifetime* (73) |
|  |  | 1. Access to resources, networks, and expertise through partnerships | | *The interaction between linkage to dominant platforms in module networks and linkage to dominant actors in resource networks will increase the likelihood of initial launch success* (63) |
|  | 1. **Suppliers & Partners** | 1. Alliance strategy in place | | *After discussing with stakeholders and the mentors, the owners focused on alliances to increase their delivery and market reach capabilities* (72) |
|  |  | 1. Collaboration with larger organizations | | *Firms that do not have strong revenue streams from existing products to support continual product improvements and market expansion should consider partnering with larger firms* (49) |
|  |  | 1. End-to-end value chain involvement | | *Scalability and rapid implementation of RPM services require social change and active collaboration between stakeholders* (51) |
|  | 1. **Competitors** | 1. Market entry timing and organizational maturity | | *To enhance their credibility, firms need to aggressively promote their first-in-market position* (49) |
|  |  | 1. Intensity of competitors in the market | | *It is the intensity of competition between Startups within the same industry* (68) |
|  |  | 1. Collaborative opportunities with competitors for mutual growth | | *Grouping interrelated companies that work in the same industrial sector and that collaborate strategically to obtain common benefits* (68) |
